# Supplementary material for: Impact of Sex and Glial Tau Expression on Heat Shock Protein Induction in a Drosophila Model of Tauopathy
Source: ACS Omega. 2025 Sep 25;10(39):45921–32. doi: 10.1021/acsomega.5c06686 (PMC12508927; doi:10.1021/acsomega.5c06686)
Supplement: Supplementary file 1 [file ao5c06686_si_001.pdf]

Supplemental Information

Impact of sex and glial tau expression on heat shock protein induction in a *Drosophila* model of tauopathy

Marguerite Whitmore[1], Maeve Coughlan[2], Martha A. Kahlson[1], Jaasiel Alvarez[1], Louisa Zebrowski[2], Kenneth J. Colodner*[2,] and Kathryn A. McMenimen*[1,2,3]

[1] Program in Biochemistry
[2] Program in Neuroscience and Behavior
[3] Department of Chemistry
Mount Holyoke College, 50 College Street, South Hadley, MA 01075

Corresponding authors*: Kathryn A. McMenimen (kamcmeni@mtholyoke.edu) and Kenneth J. Colodner (kcolodne@mtholyoke.edu)

Full western blots used to quantify protein expression in Figure 5 are shown below.

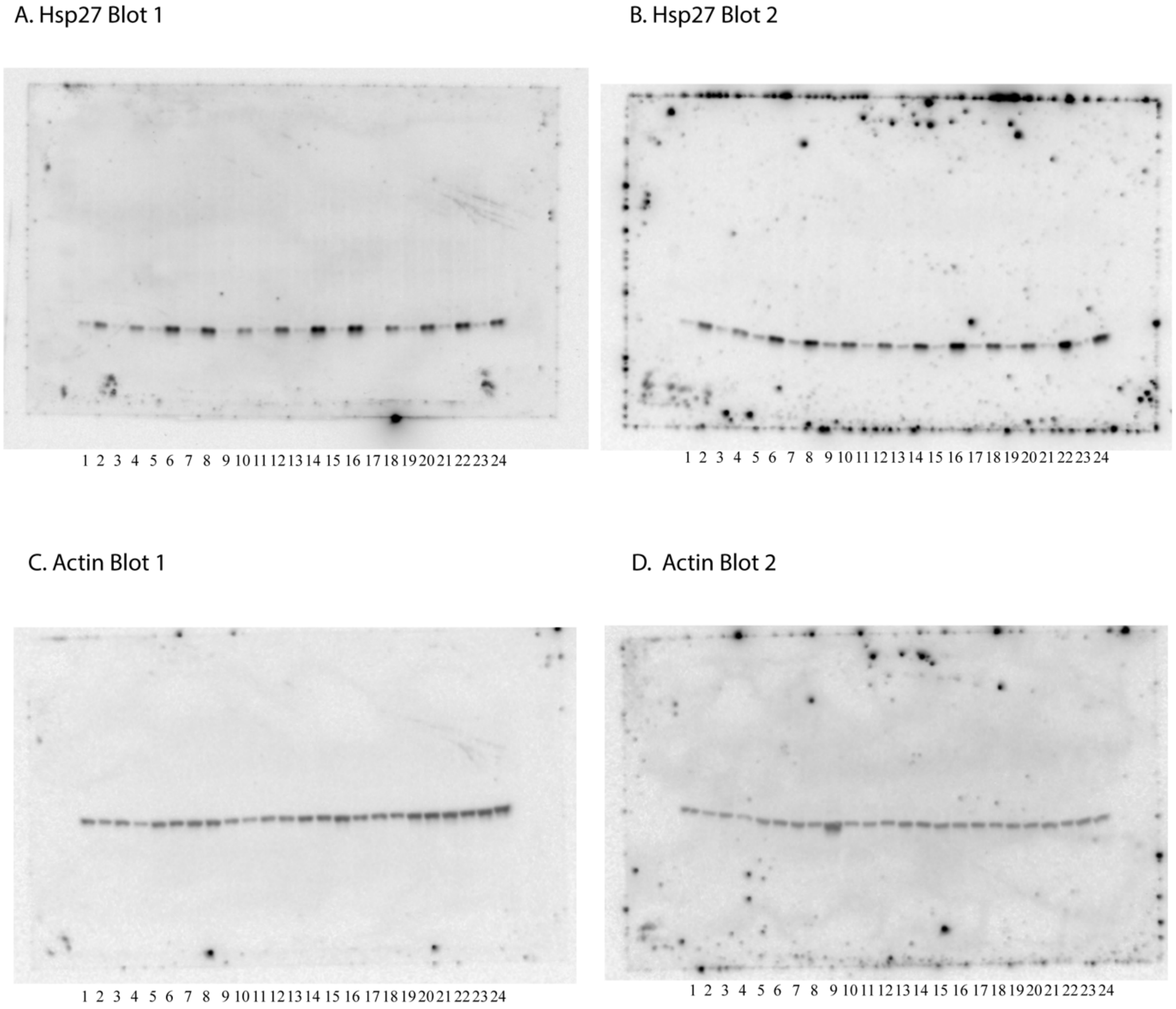

Supplemental Figure 1. Hsp27 protein expression from day 10 male and female *Drosophila* brain samples. Each blot contains 24 individual samples obtained from an individual brain. Each row is labeled from 1-24. The samples are in the following order: 1, 9, 17: control male no heat, 2, 10, 18: control male heat shock, 3, 11, 19: tau male no heat, 4, 12, 20: tau male heat shock, 5, 13, 21: control female no heat, 6, 14, 22: control female heat shock, 7, 15, 23: tau female no heat, 8, 16, 24: tau female heat shock. A. Western blot (#1) monitoring expression of Hsp27 protein in three male and three female samples for each experimental condition listed. B. Western blot (#2) monitoring expression of Hsp27 protein in three male and three female samples for each experimental condition listed. A total of 6 replicates were compiled across western blots in A and B. C. Western blot monitoring expression of Actin protein in three male and three female samples for each experimental condition listed, stripped and re-probed membrane of #1. D. Western blot monitoring expression of Actin protein in three male and three female samples for each experimental condition listed, stripped and re-probed membrane of #2. A total of 6 replicates were compiled across western blots in C and D.

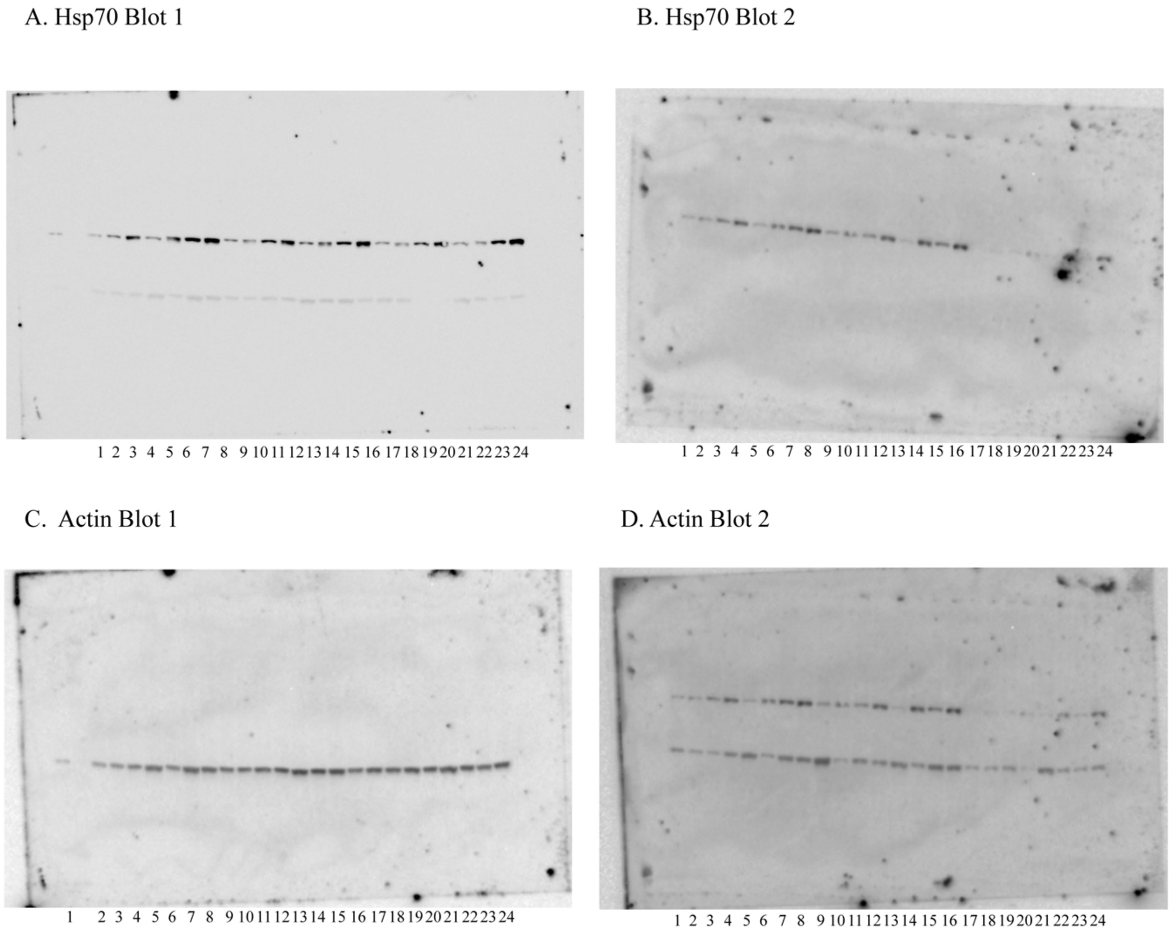

Supplemental Figure 2. Hsp70 protein expression from day 10 male and female *Drosophila* brain samples. Each blot contains 24 individual samples obtained from an individual brain. Each row is labeled from 1-24. The samples are in the following order: 1, 9, 17: control male no heat, 2, 10, 18: control male heat shock, 3, 11, 19: tau male no heat, 4, 12, 20: tau male heat shock, 5, 13, 21: control female no heat, 6, 14, 22: control female heat shock, 7, 15, 23: tau female no heat, 8, 16, 24: tau female heat shock. A. Western blot (#3) monitoring expression of Hsp70 protein in three male and three female samples for each experimental condition listed. B. Western blot (#4) monitoring expression of Hsp70 protein in three male and three female samples for each experimental condition listed. A total of 5 replicates were compiled across western blots in A and B. Note, lanes 18-24 of Western #4 were excluded from analysis due to insufficient protein transfer. C. Western blot monitoring expression of Actin protein in three male and three female samples for each experimental condition listed, stripped and re-probed membrane of #3. D. Western blot monitoring expression of Actin protein in three male and three female samples for each experimental condition listed, stripped and re-probed membrane of #4. A total of 5 replicates were compiled across western blots in C and D, as lanes 18-24 of Western #4 were excluded from analysis.
